# The association between self-perceived health status and satisfaction with healthcare services: Evidence from Armenia

**DOI:** 10.1186/s12913-016-1309-6

**Published:** 2016-02-19

**Authors:** Pavitra Paul, Mihran Hakobyan, Hannu Valtonen

**Affiliations:** Department of Health and Social Management, University of Eastern Finland (Kuopio Campus), P.O. Box 1627, 70211 Kuopio, Finland; UNICEF Armenia, 14 P. Adamyan Str., Yerevan, 0010 Armenia; Faculty of Social Sciences and Business Studies, University of Eastern Finland, 70211 Kuopio, Finland

**Keywords:** Armenia, Health system, ILCS, Satisfaction, Self-assessed health, Socioeconomic strata

## Abstract

**Background:**

Armenians very rarely seek healthcare services and, consequently experience more serious health conditions. With its ongoing reforms, Armenia is focusing on linking health system financing to the quality and volume of care provided. We examine the relationship between the perceived health status of the population and the satisfaction with healthcare services.

**Methods:**

A pooled probit model is applied to analyse three datasets (2010, 2011 and 2012) from the Integrated Living Conditions Survey (ILCS).

**Results:**

We find a strong association between self-perceived health and satisfaction with healthcare services but this association is not consistent across regions.

**Conclusions:**

The socioeconomic position of the household alone does not explain the perception of individual health status. The perceived dwelling condition and geography of residence emerged as important stressors on associations between the perceived health status of the population and the satisfaction with healthcare services. We have modelled the perceived health status and satisfaction with the healthcare services using demand side datasets. This study establishes the need to re-examine this association in a multidimensional construct.

## Background

The recognition of two main principles has led to the growth of consumerism in public policy: (1) health systems exist because of the population it is designed to serve, and (2) patients have a right to have their views taken into account when designing and evaluating the performance of health systems [1, 2]. Consequently, patient satisfaction has become a ubiquitous concept in healthcare service evaluation, because end-user satisfaction (1) reflects the perceived value that the population ascribes to the health system and (2) provides an acceptable indicator for an outcome measure that is less contentious than measuring quality of life. Studies [3, 4] suggesting that satisfied patients are more likely to follow planned case management and make better use of health services indicate that satisfaction has an independent influence on the effectiveness of care.

Patient compliance, the use of health services, continuity of care, and self-perceived health status have been found to be associated with satisfaction with healthcare services [5–9]. Access to care and the responsiveness (interpersonal interaction) and appropriateness (technical quality) of healthcare services have been found to account for nearly two-thirds of the variance in overall patient satisfaction [10]. Satisfaction may have an independent influence on the effectiveness of care if the satisfied patients are more likely to follow planned care and make better use of healthcare services [3, 4]. The concern that there is a differential perception [11, 12] about health status and consequent opinion about healthcare services between different socioeconomic strata (SES) of the population is central to a policy that aims to achieve distributional efficiency for health systems. These observations have intensified interest in examining consumer satisfaction with healthcare services [13].

There are varying results in the literature concerning causality – as opposed to association – between health status and satisfaction with healthcare services. It is known from earlier research [9, 14] that self-perceived health status is associated with satisfaction with services. Satisfaction with healthcare services is seen as both a consequence [15] and a determinant [16] of health status. Satisfaction can be defined as an ‘evaluation based on the fulfilment of expectations’ [17]. Thus, satisfaction may reflect not only the perceived quality or performance of the healthcare services but also the health status of the individual. Empirical evidence typically suggests that populations with better health tend to report greater satisfaction with the healthcare services used [9]. Furthermore, evidence also suggests that dissatisfaction with the healthcare system may, in large part, be a manifestation of dissatisfaction with life [18]. Hong and Barber [14] have argued that patient satisfaction is a function of patient well-being and other personal characteristics unrelated to healthcare services. People who perceive themselves to be in poorer health may report lower patient satisfaction because they associate their poorer health with the healthcare they receive. Conversely, individuals who feel well may project that sense of wellness onto their health system environment and report being satisfied with it. While Williams and Calnan [19] and Roberts et al. ([18 p. 379 ]) found no significant correlation between perceived health status and satisfaction with healthcare, many studies [20–26] have reported that dissatisfaction with healthcare services is associated with perceived poor health status. Furthermore, it has been claimed that the positive association between patient satisfaction and health status is more likely to represent a tendency among healthier patients to report greater satisfaction with healthcare services rather than a tendency among patients who improve following interaction with the health system to report greater satisfaction [27, 28]. Studies ([21], p 226, [29]) have also reported health status as a causal determinant of satisfaction with the healthcare services used, though the causal process underlying this relationship is not clear. However, some authors argue that satisfaction with specific aspects of healthcare services contributes independently to either mental or physical health status [30].

Health status and satisfaction with healthcare are not regarded as multidimensional constructs [31–33], but on the other hand, the association between health status and satisfaction with healthcare services is better assessed after controlling for other confounding factors, such as age ([33], p 1091) and education [14, 34]. The effect of gender on the association between self-perceived health status and satisfaction with healthcare services is inconsistent [21, 35–38].

Armenians very rarely^1^ seek ambulatory services and, as a result, experience more serious health conditions that warrant in-patient care. The Primary Health Care Reform (PHCR) project (2006) revealed that in Yerevan, 44.5 % of residents needing medical care went directly to pharmacies instead of visiting a doctor and getting a proper prescription. This was done in part to avoid making out-of-pocket payments to doctors who require fees for consultations [39].

Sharp differences in the financial capacities of communities are reflected in the development and maintenance of the community’s socio-economic infrastructures, as well as in unequal capacities for the delivery of community services. Small communities, often with limited budgets and low revenues, cannot address not their development issues, let alone meet the population’s basic social needs. This situation results in significant differences across communities as regards human development [40]. The Republic of Armenia is focusing on linking health system financing to the quality and volume of care provided [41]. A co-payment mechanism was introduced in March 2011 to improve the quality of service through wage increases for healthcare professionals and the improved provision of technology and supplies; however, the amount of the co-payment is substantial and unaffordable for the majority of the Armenians [42]. Compared to 2009, the number of people who in 2012 did not access needed healthcare services increased [43]. All these observations seem to indicate that access to healthcare is constrained in Armenia.

Typically, satisfaction with the health system has been treated as either a single broad domain reflecting general satisfaction with healthcare services [9, 44] or interpreted as a linkage between poor self-perceived health status and dissatisfaction with healthcare services [9]. Although both hypotheses refer to satisfaction with healthcare services, the two models lead to distinctly different expectations with regard to specific domains of satisfaction with the health system. The key issue that emerges is the extent to which satisfaction with healthcare is determined by factors other than the healthcare services received by the individual.

From a health policy and equity point of view, it is important to know the regional differences in the association between self-perceived health, utilization of health services, and satisfaction with the health services. However, it may be even more important to know the associated factors and the factors causing the differences. In this study, we (1) measure the in-country distribution of age- and gender-standardized perceived health status across the socioeconomic groups of the population, and (2) examine the relationship between the perceived health status of the population, the satisfaction with healthcare services, and the use of healthcare services. Although studies have established a relationship between self-perceived health status and satisfaction with healthcare services [5, 9, 14], this is the first study to use survey datasets from a former USSR country for examining the effect of self-perceived health status on the satisfaction with healthcare services. We use recent Armenian survey datasets from 2010, 2011 and 2012 in our analyses.

### Characteristics of the Armenian health system

During the Soviet era, healthcare coverage was universal and healthcare services were formally free at the point of use. The overall management of the system was limited by a lack of both resources and performance incentives. The emphasis was on secondary care and in-patient treatment over primary care and out-patient treatment. The collapse of the Soviet Union created a unique opportunity for health system reforms in Armenia. These included the Health Care Law (1996), the introduction of the Basic Benefit Package (1997), and the establishment of the State Health Agency (1998). Health system reforms in Armenia have included (1) the prioritization of primary healthcare, (2) optimization of the hospital network and a reduction in hospital beds, (3) decentralization of service provisions to regional and local governments, (4) service privatization, and (5) efforts to improve health service use through quality of care [45]. The State Health Agency (SHA) is responsible for purchasing services from providers according to the Basic Benefit Package (BBP) and the principles of quality assurance. The spending of public budget funds by the Ministry of Finance to purchase services from healthcare providers is decided by contracts between the SHA and the service providers. In general, SHA payments to the providers are less than the costs, and healthcare facilities collect sizable out-of-pocket payments.

Primary care budgets are inadequate to cover many guaranteed benefits, even for socially vulnerable population groups [46]. In 2012, total per capita expenditure on healthcare was International $299 (Government expenditure $125.20) and public expenditure on the health system as a percentage of GDP was 1.9 % [47]. The physical conditions in health posts and polyclinics are often poor [42]. Further, Armenian statistics [48] reveal that there are relatively large differences in the deployment of human resources for healthcare services between regions. For example, the ratio of nurses in 2008 varied from a low of 397 (per 100,000 citizens) in Ararat to a high of 548 in Vayots Dzor, and the ratio of physicians varied from a high of 321 in Yerevan to a low of 139 in Armavir. The role of private healthcare facilities is becoming increasingly apparent in Armenia’s healthcare framework ([42], p 12).

The health system reforms provide patients with the right to choose their primary care physicians but limit rights to access direct specialist care unless specialist services are paid for directly, i.e. bypassing referral from primary care physicians. The share of informal payments has been highest (91 %) among the former USSR countries, and access to healthcare services has become increasingly dependent on the household’s ability to afford informal payments to providers [49]. The low per capita income and the existence of an informal shadow economy are posing considerable challenges in the development of health insurance coverage. A co-payment mechanism was introduced in 2011 to increase the salaries of employees at the health facility-level, and to improve service quality by increasing the provision of medicine and equipment. From the individuals’ perspective, one important barrier to the consumption of healthcare services is the system of regionally varying out-of-pocket payments. In 2012, only 10.5 % of the beneficiaries of the poverty alleviation programme had the right to use medical services guaranteed by the state, and increased state financing of the health system in recent years has not significantly decreased the amount of out-of-pocket payments made by citizens [50]. In 2009, 18 % of the population was entitled to the basic benefit package; however, only 49 % of the eligible population used healthcare services under BBP – this figure breaks down as 77 % of the extremely poor, 51 % of the poor, and 6 % of the non-poor ([42], p 16). Despite significant health sector reform since independence, inequities in access to care remain obvious. For example, in 1999, the utilization of government-financed healthcare services by the richest 20 % of the population was three times higher than that of the poorest 20 % [51].

## Methods

We used three cross-sectional survey datasets from the Integrated Living Condition Survey (ILCS), Armenia from three consecutive years (2010, 2011, and 2012). The three cross-sectional survey datasets comprise a total of 83,511 individuals nested in 20,928 households. The ILCS (datasets are available at http://www.armstat.am/en/?nid=378) was conducted by the National Statistical Service (NSS) of the Republic of Armenia. All regions as well as all urban and rural settlements were included in the sample population according to the share of households based in those settlements as a percentage of the total households in the country. Table 1 presents the characteristics of the survey population and healthcare service users for each of the three years.

**Table 1:** Characteristics of the survey population and healthcare service users for each year

	2012		2011		2010	
Respondents	Survey population	Healthcare Service user	Survey population	Healthcare Service user	Survey population	Healthcare Service user
Number of households	5,184	976	7,872	1,567	7,872	1,849
Number of individuals	20,134	1,185 (5.89 %)	31,024	1,949 (6.28 %)	32,353	2,293 (7.09 %)
Gender distribution (%)	Male: 48.19	Male:35.11	Male: 48.75	Male:39.35	Male: 48.69	Male: 37.85
	Female: 51.81	Female: 64.89	Female: 51.25	Female: 60.65	Female: 51.31	Female: 62.15
*Age group (years)*	(%)	(%)	(%)	(%)	(%)	(%)
15–30	44.03	27.34	44.66	28.37	47.14	29.26
31–44	17.14	8.61	16.43	10.93	16.26	12.25
45–60	22.4	27.93	22.98	26.53	22.16	30.0
≥61	16.42	36.12	15.94	34.17	14.45	28.48
*Education*	(%)	(%)	(%)	(%)	(%)	(%)
Below secondary school	20.8	19.01	22.57	22.12	23.66	20.8
Secondary school (incl. vocational)	39.11	38.02	40.82	38.74	40.88	38.66
College and above	40.09	42.97	36.61	39.14	35.46	40.54
*Settlement of residence*	(%)	(%)	(%)	(%)	(%)	(%)
Urban	64.33	69.45	53.59	56.03	53.29	56.57
Rural	35.67	30.55	46.41	43.97	46.71	43.44
Distribution by region (%)	(n = 20,134)	(n = 1,185)	(n = 31,024)	(n = 1,949)	(n = 32,353)	(n = 2,293)
Yerevan	26.31	35.19	16.48	22.83	16.85	20.98
Aragatsotn	5.64	12.24	6.83	15.6	7.05	13.13
Ararat	8.47	6.75	9.73	5.54	9.82	7.68
Armavir	9.17	4.47	10.31	4.36	10.01	7.28
Gegharkunik	6.88	2.03	7.97	1.8	8.45	1.79
Kotayk	7.02	5.15	11.46	11.85	10.86	9.68
Lori	10.04	13.0	8.96	8.77	8.86	10.29
Shirak	8.16	3.54	10.15	4.67	10.16	5.32
Sjunik	5.57	7.0	5.64	11.75	5.56	11.6
Tavush	6.0	5.91	7.29	8.11	7.26	6.93
Vayots Dzor	6.72	4.73	5.17	4.72	5.12	5.32
Distribution by SES^a^ (%)						
Poorest	20.02	19.07	20.18	18.93	20.0	18.27
Poor	20	26.24	19.83	23.7	20.0	25.03
Middle	19.99	18.57	20.03	20.73	20.17	21.72
Affluent	20.05	18.31	20.1	18.37	20.37	15.39
Richest	19.95	17.81	19.87	18.27	19.46	19.58
*Self-perceived health status* ^*b*^ (%)						
Excellent	6.86	2.03	6.67	1.33	7.43	1.79
Good	40.83	12.66	39.91	13.03	38.62	13.08
Average	41.16	32.24	42.98	37.92	42.87	38.99
Poor	10.43	48.61	9.81	44.54	10.22	41.91
Worst	0.73	4.47	0.63	3.18	0.86	4.23
Diagnosed with high blood pressure (%)	38.99	38.99	40.23	40.23	30.79	30.79
*Use of healthcare services* ^*c*^ *(%)*	6.39	-	6.79	-	7.65	-
BBP coverage^d^ (%)	6.11	18.48	6.62	19.19	5.95	20.15
*Poverty alleviation coverage* ^*e*^	13.42	13.92	10.64	11.9	13.77	14.3
*Perceived economic strata* ^*f*^ *(%)*						
Well-off	3.91	2.69	2.92	2.03	4.0	3.34
Middle	79.74	79.33	81.19	81.31	80.3	82.56
Poor	16.35	17.98	15.89	16.66	15.7	14.1
*Forwent medical care* ^*g*^ *(%)*						
explicit reason: affordability	5.15	-	4.83	-	5.52	-
explicit reason: distance	0.05	-	0.15	-	0.17	-
*Level of satisfaction with healthcare systems* ^*h*^ *(%)*						
Satisfied	-	34.26	-	39.15	-	36.15
Not satisfied	-	65.74	-	60.85	-	63.85

^a^Constructed socio-economic strata (SES) by quintiles: poorest, poor (=2^nd^ poorest), middle, affluent (=2^nd^ richest), and richest

^b^Five group of respondents: very good; good; neither good nor bad; bad, and very bad

^c^Use of healthcare services: use of medical services from a family doctor, ambulatory clinic, polyclinic or village health centre within the preceding 30 days of responding to the enumerator

^d^Basic Benefit Package (2006) is a publicly funded package that specifies services that are either fully funded (for certain socially vulnerable groups, such as those living in poverty) or partly covered; these services include primary care, maternity services, sanitary-epidemiological services and treatment for around 200 socially significant diseases. Emergency services are also covered, but with some co-payments for all but the socially vulnerable groups

^e^Poverty alleviation coverage: Introduced in 1999 – a monthly cash benefit to very poor households using a proxy means testing mechanism (PMT) consisting of social risk category, number of family members incapable of working, place of residence, housing condition, private business, and family income

^f^Response to the question: ‘Which category do you think your family belongs to?’

^g^Forwent medical care: has not sought medical care, when asked the reasons for not seeking medical care within the preceding 30 days of responding to the enumerator

^h^Opinion about healthcare services by the respondents who used healthcare services

The satisfaction with the services was asked only of those survey respondents who had used healthcare services (‘healthcare services user’ in Table 1) in the 30 days before each survey period. The three cross sectional survey datasets together comprise a total of 5,427 healthcare service users. Within the survey population, the number of respondents who had used healthcare services was less in 2012 than in 2010, though the dissatisfaction amongst the users of healthcare services increased by around 3 % in 2012. A higher percentage of the population with poor or the worst self-perceived health (SAH) were found in the user of healthcare services dataset for all three years. Female respondents and the respondents with tertiary education (college or higher) from urban settlements and from poor SES were relatively more represented in the healthcare service user datasets than in the survey population. The distribution of respondents on different perceived economic strata and the distribution of those who forwent medical care (due to affordability issues or distance) were found to be almost same in all three years of study period.

When using self-perceived health (SAH) as our explanatory variable, we wanted to minimize the loss of information and some of the cross-sample variation that comes with selecting an arbitrary cut-off point. Therefore, we used SAH as a continuous variable (1 = excellent to 5 = worst) instead of a dichotomization with a cut-off point [52].

In the data, there was no income variable available that could be used to determine the household’s position in the socio-economic strata (SES) classification. To measure SES, studies have used variables, such as agricultural land ownership [53], farm animals and ownership of the place of residence ([12], p 363), and crowding [54]. Houweling et al. [55] found that inclusion of a sanitation facility variable increased inequality amongst households. Lindelow [56] concluded that the inclusion of infrastructure variables increased SES inequality in healthcare service use.

We constructed an SES index [57–59] by applying principal component analysis (PCA) using a set of variables measuring living conditions (Table 2). The PCA approach in constructing SES indices has allowed us to overcome the limitations of (1) income-based approaches [60, 61] and also (2) consumption-based approaches ([53], p 116). We have taken only those variables that were found to be ‘meritorious and marvelous’ [62] in PCA. The overall Kaiser-Meyer-Olkin statistic for the chosen variables is 0.88. We have also examined the squared multiple correlations of the variables to validate the significance of these variables for inclusion in PCA. The inclusion of a sufficiently broad range of variables and also a continuous variable (crowding) has enabled us to construct the SES indices without the problems of truncation^2^ ([60], p 9). We constructed a common SES indicator for the rural and the urban population, and we used the weighted sum of the standardized variables to obtain the SES score. Finally, households were grouped into quintiles reflecting their place in the different SES groups.

**Table 2:** Definition of variables

Name of the variable	Description of the variable
Satisfaction with healthcare services:	1 = satisfied, 0 = not satisfied
Explanatory variable	
Self-perceived health (SAH):	1 = Excellent, 2 = good, 3 = average, 4 = poor, 5 = worst
Confounding factors	
Socio-economic strata (SES):	quintile 1 = poorest, 2 = poor, 3 = middle group, 4 = affluent, 5 = richest
	Living standard (condition) variables in the survey used in principal component analysis (PCA) to create the SES measure: type of dwelling unit (1 = detached house, 0 = other); ownership of the residence (1 = own, 0 = don’t own); connected to the centralized sanitary (sewerage) services (1 = yes, 0 = no); crowding (square metre living space available per household member); toilet outside the dwelling unit (1 = yes, 0 = no); access to a computer with internet at home (1 = yes, 0 = no); production on household owned agricultural land (1 = yes, 0 = no); and ownership of livestock/cattle (1 = yes, 0 = no)
Presence of chronic disease:	Diagnosed with high blood pressure (1 = yes, 0 = no)
Use of healthcare services:	(1 = yes, 0 = no)
Coverage with BBP (Basic benefit package):	1 = yes, 2 = no
Recipient of poverty benefit: 1 = yes, 2 = no	
Settlement of residence:	capital city, urban, rural
Dwelling condition (a self-perceived expression as a response to the question ‘please evaluate your housing condition’):	1 = good; 2 = satisfactory, 3 = bad
Used medical services in the last thirty days:	1 = once, 2 = twice, 3 = more than twice
Education:	1 = below secondary school, 2 = secondary school (including vocational training), 3 = college and above
Demography:	age (15 years and above) and gender (1 = male and 0 = female)
Geography of residence:	Region (Capital city, Yerevan; other regions are: Aragatsotn, Ararat, Armavir, Gegharkunik, Kotayk, Lori, Shirak, Sjunik, Tavush and Vayots Dzor)
Year of survey: 2010, 2011, and 2012.	

After constructing the SES indices, we measured age- and gender-standardized [63] self-perceived health status (SAH) for each SES quintile across regions of the country.

$$ {\widehat{y}}_i^x=\widehat{\alpha}+{\displaystyle {\sum}_j\widehat{\beta_j}{x}_{ji}}+{\displaystyle {\sum}_k{\widehat{\gamma}}_k{\overset{\mathit{\hbox{'}}}{z}}_k}\kern0.37em \mathrm{where}, $$

ordinary least squares (OLS) parameter estimates $\left(\widehat{\alpha},\;\widehat{\beta}j,\widehat{y}k\right)$ individual values of the confounding variables (*x*_*ji*_) and sample means of non-confounding variables $\left(\overset{\mathit{\hbox{'}}}{{\mathrm{Z}}_k}\right)$ are then used to obtain *ŷ*_*i*_^*x*^ = *x* -expected (predicted) value of SAH.

Estimates of indirectly standardized SAH, *ŷ*_*i*_^*IS*^, are then derived from the difference between actual and *x*-expected health, plus the overall sample mean $\left(\overset{\mathit{\hbox{'}}}{y}\right)$ for the quintile for the year.

$$ {\widehat{y}}_i^{IS}={y}_i-{\widehat{y}}_i^x+\overset{\mathit{\hbox{'}}}{y} $$

The distribution of *ŷ*_*i*_^*IS*^ (across SES) is the distribution of SAH that is expected to be observed, irrespective of differences in the distribution of *x*s (age and gender) across SES.

In the final analysis set, we used a pooled probit model to find the relationship between standardized SAH and opinion of the healthcare services.

$$ {l}_{it}=\alpha +\beta {\widehat{Y}}_{it}^{IS}+{\varepsilon}_{it},i=1,\dots \dots, N;t=1,\dots, T. $$

*l*_*it*_*is the latent variable that represents the observed level of satisfaction with health services;*

*Ŷ*_*it*_^*IS*^*is the demography adjusted and SES quintile-specific health.*

We used two sets of variables: a) Outcome variable = satisfaction with healthcare services, Predictor variable = standardized self-perceived health (SAH); and b) confounding factors: SES, education, and settlement of residence.

In our data, the latent outcome *l*_*it*_ is not observed. Instead, we observed a binary indicator of the category in which the latent indicator falls (*l*_*it*_).

The observation mechanism is:

$$ \bullet {l}_{it}=1, if{l}_{it}>0, $$

$$ \bullet {l}_{it}=0, otherwise. $$

Here, a pooled model assumes that the error term ${\varepsilon}_{it}$ is distributed as N (0,1). Though the pooled models do not explicitly take account of the panel nature of the dataset, the estimator is a consistent estimator of the parameters of interest. The pooled estimator does not require the regressors to be strictly exogenous and it can accommodate predetermined variables [64]. This makes the estimator more robust in comparison to a random effects specification, where strict exogeneity is assumed. We have also estimated marginal effects (elasticities) at the means of the independent variables on the probability that the dependent variable takes the value 1.

The models were tested for multicollinearity and all the values of the variance inflation factor (VIF) were less than 5 for all the variables used. The Cook-Weisberg test for heteroscedasticity reported a homogeneous variance and the Ramsey RESET test did not indicate any specification problems.

Additionally, we tested for a selection effect on the healthcare user sample. For example, it might be the case that, ceteris paribus, dissatisfied people use fewer services than satisfied people. However, we did not find this kind of selection effect, i.e. the effect of satisfaction on healthcare service use.

## Results

The variations of service use and satisfaction between the regions were quite large (Table 3). The highest percentage (approx. 17 %) of healthcare service use was in Aragatsotn, with 41 % of respondents satisfied, while the highest percentage (approx. 44 %) of satisfied respondents were in Kotayk, where about 6 % of respondents had used the healthcare service. The relatively lowest percentage of service use was in Gegharkunik. Barring Kotayk, the mean standardized perceived health status reflected a relatively better health status for those service users who were satisfied. The difference in mean standardized perceived health status between the healthcare service users who were satisfied and those who were not was highest in Armavir, Shirak, and Tavush, indicating a better self-perceived health status for satisfied healthcare service users.

**Table 3:** Distribution of healthcare service users by service use, satisfaction with healthcare services, and standardized perceived heath status (mean) by region

Region	Service used (%)	Satisfied (%)	Standardized perceived health status - mean, if satisfied
Yerevan (n = 1,343)	8.79	38.05	2.9 (3.4)
Aragatsotn (n = 750)	15.66	40.8	3.1 (3.3)
Ararat (n = 364)	5.01	27.2	3.1 (3.5)
Armavir (n = 305)	3.86	39.02	2.5 (3.2)
Gegharkunik (n = 100)	1.67	38.0	2.7 (3.0)
Kotayk (n = 514)	6.34	43.97	3.3 (3.1)
Lori (n = 561)	8.17	32.26	2.8 (3.2)
Shirak (n = 255)	3.60	25.88	2.7 (3.4)
Sjunik (n = 578)	13.15	43.43	2.8 (3.2)
Tavush (n = 387)	7.15	24.81	2.8 (3.5)
Vayots Dzor (n = 270)	6.47	38.89	3.1 (3.5)
*Total (n = 5,427)*	7.03	36.82	2.9 (3.3)

Figures in parentheses indicate the standardized self-perceived health status if not satisfied

For all the regions barring Gegharkunik, Cramer’s V was more than 0.30 (Table 4), indicating a strong relationship between satisfaction with healthcare service use and self-perceived health status. A Cramer’s V of 0.24 for Gegharkunik suggests a moderate relationship between satisfaction with healthcare service use and self-perceived health status. The distributions of self-perceived health status amongst the healthcare service users were not uniform across the regions. Armavir had the largest proportion of healthcare service users with a self-perceived health status of excellent, while Vayots Dzor had the largest proportion of healthcare service users with a self-perceived health status of below average.

**Table 4:** Distribution of satisfied healthcare service users and self-perceived health status across regions

Region with healthcare service users	Self-perceived health status (%)					Association between self-perceived health status and satisfaction with healthcare services (Cramer's V)
	Excellent	Good	Average	Poor	Worst	
Yerevan (n = 1,343)	1.41	16.08	32.46	44.45	5.58	0.32
Aragatsotn (n = 750)	0.27	3.60	50.27	45.60	0.26	0.38
Ararat (n = 364)	1.10	7.14	32.97	50.27	8.52	0.34
Armavir (n = 305)	9.51	11.15	38.03	36.72	4.59	0.33
Gegharkunik (n = 100)	8.00	17.00	41.00	27.00	7.00	0.24
Kotayk (n = 514)	1.75	8.17	40.47	47.08	2.53	0.45
Lori (n = 561)	0.71	15.86	43.49	37.08	2.85	0.30
Shirak (n = 255)	3.92	16.08	20.39	53.73	5.88	0.36
Sjunik (n = 578)	0.35	22.84	41.87	34.60	0.35	0.31
Tavush (n = 387)	1.03	12.14	27.65	53.49	5.68	0.38
Vayots Dzor (n = 270)	0.00	12.22	26.67	55.56	5.56	0.31
*Total (n = 5,427)*	1.68	12.97	37.13	44.32	3.91	0.34

We found an association between the SES of the respondents and the standardized perceived health status (Table 5).The standardized perceived health status varied with chronic disease (diagnosed with high blood pressure), between the SES groups, dwelling conditions, coverage with the basic benefit package (BBP), being a beneficiary of poverty alleviation measures, and region of residence. Again, over the years, the direction of this change was not uniform. The association with SES was u-shaped; when compared to the poorest SES quintile, the second poorest quintile had worse perceived health, but the three higher quintiles did not differ statistically significantly from the lowest SES group. The other living standard indicators (dwelling, BBP coverage, and poverty alleviation coverage) explained the differences in the perceived health status, as could be expected; health status was worse with poorer living conditions [65]. After all the obvious factors were taken into account, however, regional differences in the self-perceived health status were evident.

**Table 5:** Ordinary least square model for standardized self-perceived health (1 = excellent, 5 = worst)

Variables	β coefficient	95 % confidence interval	
Diagnosed high blood pressure	0.106***	0.068	0.144
Education (comparison group = below secondary school)			
Secondary school (incl. vocational)	0.020	-0.031	0.071
College and above	-0.048	-0.100	0.005
SES (comparison group = poorest quintile)			
Poor	0.088***	0.027	0.149
Middle	0.054	-0.013	0.121
Affluent	-0.004	-0.092	0.084
Richest	-0.024	-0.116	0.069
Dwelling condition (comparison group = good)			
Satisfactory	0.063*	0.007	0.118
Bad	0.253***	0.187	0.320
BBP coverage	0.211***	0.162	0.261
Poverty benefit beneficiary	0.106***	0.050	0.162
Settlement (comparison group = capital city)			
Urban	-0.030	-0.121	0.062
Rural	0.063	-0.049	0.176
Year (comparison = 2010)			
2011	-0.017	-0.062	0.027
2012	0.006	-0.044	0.056
Region (comparison group = Capital city, Yerevan)			
Aragatsotn	0.001	-0.088	0.091
Ararat	0.066	-0.038	0.170
Armavir	-0.218**	-0.344	-0.093
Gegharkunik	-0.389***	-0.565	-0.213
Kotayk	-0.092	-0.189	0.004
Lori	-0.203***	-0.297	-0.110
Shirak	-0.069	-0.195	0.057
Sjunik	-0.216***	-0.304	-0.127
Tavush	0.005	-0.098	0.107
Vayots Dzor	0.076	-0.030	0.183
Intercept	3.05***	2.959	3.148
R^2^ = 0.082			
F (p = 0.000) = 18.34			
*N* = 5,427			

Legend: *p < 0.05;**p < 0.01;***p < 0.001

Table 6 shows the model explaining satisfaction (as a binary variable) with healthcare service. In line with earlier studies [27, 28], we found a significant association between satisfaction and standardized self-perceived health status. In addition, we also found an association of use (as percentage of the population) and satisfaction. The confounding variables, i.e. age, chronic disease (diagnosed with high blood pressure), education, service use, dwelling condition, and region of residence were also associated with satisfaction. Gender, age and gender interaction, SES, and BBP coverage did not have a statistically significant association with satisfaction. However, we found a significant association of age and gender-standardized self-perceived health status with SES, dwelling condition, coverage with the basic benefit package (BBP), being a beneficiary of poverty alleviation measures, and region of residence (Table 5).

**Table 6:** Pooled probit model for satisfaction with the healthcare services

	Effect on unobserved (latent) opinion about healthcare services [${l}_{it}$]	95 % confidence Interval		Elasticity
Standardized self-perceived health (min. = best; max. = worst)	-0.477***	-0.537	-0.416	-0.17***
Gender (1 = male)	0.130	-0.074	0.333	0.05
Age	-0.052***	-0.062	-0.041	-0.02***
Age squared	0.000***	0.000	0.000	0.00***
Male*Age	0.002	-0.002	0.005	0.00
Diagnosed high blood pressure	-0.094*	-0.188	-0.001	-0.03*
Education (comparison group = below secondary school)	0.124	-0.001	0.249	0.04
Secondary school (incl. vocational)	0.184**	0.052	0.315	0.07**
College and aboverow				
Use of healthcare services	0.073***	0.033	0.113	0.03***
SES (comparison group = poorest quintile)				
Poor	-0.061	-0.186	0.063	-0.02
Middle	0.024	-0.116	0.164	0.01
Affluent	0.017	-0.168	0.202	0.01
Richest	0.058	-0.140	0.256	0.02
Dwelling condition (comparison group = good)				
Satisfactory	-0.115*	-0.234	0.003	-0.04*
Bad	-0.238***	-0.378	-0.098	-0.08***
BBP coverage	0.098	-0.007	0.203	0.03
Poverty benefit beneficiary	-0.105	-0.228	0.018	-0.04
Settlement (comparison group = capital city)				
Urban	0.097	-0.096	0.290	0.03
Rural	0.060	-0.182	0.301	0.02
Year (comparison = 2010)				
2011	0.124*	0.023	0.226	0.04*
2012	0.135*	0.014	0.257	0.05*
Region (comparison group = Capital city, Yerevan)				
Aragatsotn	-0.501**	-0.835	-0.167	-0.16***
Ararat	0.043	-0.230	0.316	0.02
Armavir	0.242	-0.061	0.545	0.09
Gegharkunik	0.377	-0.054	0.808	0.14
Kotayk	0.435***	0.213	0.658	0.16***
Lori	-0.277**	-0.486	-0.067	-0.09**
Shirak	-0.047	-0.382	0.288	-0.02
Sjunik	-0.531***	-0.811	-0.252	-0.16***
Tavush	-0.346**	-0.590	-0.103	-0.11***
Vayots Dzor	0.310*	0.059	0.562	0.12*
Intercept	1.798***	1.225	2.371	
Pseudo R^2^	0.17			
Wald chi^2^ (p)	886.46 (0.000)			
Elasticity at the mean of each variable				0.31
*N*	5,427			

Legend: *p < 0.05;**p < 0.01;***p < 0.001

An estimation of marginal effect (elasticity) with all other variables as constants shows that a one unit improvement of the mean standardized self-perceived health status would increase the probability of being satisfied by 0.17 % and one unit more use of health services would increase the probability of increased satisfaction by 3 %. Conversely, every additional year of increasing age and a shift towards the ‘bad’ dwelling condition pull the probability of expressing a ‘satisfied’ opinion down by 2 % and 8 % respectively (Table 6). The slope of the probability curve for ‘satisfied’ health service users, holding all the explanatory variables constant at the respective mean, was 0.31. By the marginal effect scores, the largest associations with satisfaction were with standardized self-perceived health status (-0.17) and with three of the Armenian regions (Aragatsotn: -0.16, Kotayk: -0.16 and Sjunik: -0.16) after we have taken into account the association of gender, the living standard indicators, and service use.

Two potentially important pieces of evidence for health policy were discovered. Firstly, in some Armenian regions (Ararat, Lori, Shirak, and Tavush) satisfaction was lower than in the other regions; self -perceived health status (Table 5) was at the same level as Yerevan in six regions (Aragatsotn, Ararat, Kotayk, Shirak, Tavush, and Vayots Dzor) and higher than in Yerevan in the remaining four regions, i.e. Armavir, Gegharkunik, Lori, and Sjunik. Secondly, beyond self-perceived health status, the extent of service use and perceived dwelling condition (Table 6) were important predictors of satisfaction with healthcare services in Armenia.

## Discussion

We measured the in-country distribution of age- and gender-standardized perceived health status across socioeconomic groups of the population. There is a strong association between SES and standardized perceived health status. The changes in the standardized perceived health status across SES over the three observation years were different in each of the regions.

Consistent with the findings of previous studies [23, 30, 33], we found a strong association between perceived health status and expressed satisfaction during the study period. The distribution of self-perceived health status associated with the use of health services was differentially associated across regions – our findings could not establish any consistent direction of relationship between use of healthcare services and self-perceived health status (Table 4). SES has a u-shaped association with age and gender standardized self-perceived health status when compared to the poorest quintiles. However, after standardization and taking the living standard variables into account, there are still significant regional differences in the perceived health level, with several regions having a higher perceived health status than in the capital city, Yerevan.

The distribution of SES and standardized mean perceived health status by SES for each region was found to be a contentious issue. The differences between the regions in the standardized mean perceived health status are difficult to explain, but can be explained by the findings of Tonoyan and Muradyan ([42], p 16), who argue that most of the population prefer the ‘ostrich method’: ‘Better not to know about our disease, than to know about it and not be able to treat it because of lack of access to paid or more expensive services and drugs’.

When we examined the relationship between the perceived health status of the population and their opinion of the healthcare services, we found a strong association between standardized perceived health status and satisfaction with healthcare services, and also between the use of healthcare services and satisfaction. However, the association between satisfaction and self-perceived health status appeared stronger (as measured by elasticities) than the association between use and satisfaction. Further, it was evident that increased use (i.e. increased percentage of the population using healthcare services) had a more positive contribution to satisfaction with the healthcare services. The elasticity of standardized perceived health status in defining opinion on healthcare services was statistically significant (-0.17), and the same was true with the use of healthcare services (0.03). The estimated effect of age, being diagnosed with high blood pressure, services use, and perceived dwelling condition were found to have a negative effect on satisfaction with the healthcare services.

Consistent with earlier studies [35, 36], increasing age significantly reduced the probability of having expressing satisfaction with the healthcare services. In line with previous findings [14, 34], respondents who completed higher education (college and above) were more satisfied with the healthcare services. There was no interaction effect between gender and age. Good dwelling conditions increased the likelihood of satisfaction with healthcare services – a variable not typically considered for inclusion in studies of satisfaction with healthcare services. The combined responsiveness at the mean of all the variables in having a ‘satisfied’ opinion was 0.31 – the satisfaction varies between the regions after the effects of all other variables have been taken into account.

The regional differences in perceived health status and satisfaction with the services could not fully be explained with the available datasets. Our data reflected the demand side of healthcare service use. Further, an explicit distribution of respondents by villages, border communities, and mountain communities would be needed to explain the effect of regions on satisfaction with healthcare services.

## Conclusion

This study provides evidence that in Armenia, inequalities in the perceived health status of the population are determined by geographical and socioeconomic factors. This study demonstrates (1) wide in-country variations in age and gender standardized the self-perceived health status of Armenians, and (2) the relationship between opinion about the healthcare services and the perceived health status of the population is highly contextual and often conditioned by the socio-cultural environment of the population. We also found evidence that it is not the objectively determined socioeconomic position but the perceived dwelling condition of the individual is an important determinant for having the expressed satisfaction with the healthcare services. In addition, the effect of increased service use also exists when opinion about the healthcare services finds a meaningful reflection. These observations suggest that opinions about health system in particular – the discrete dimensions of satisfaction with healthcare services – require cautious interpretation when comparing nations on the basis of health systems from the perspective of the citizens.

For evidence informed health policy development, the unfolding of supply-side factors in the Armenian health system is important in reducing in-country health disparities. Though health policy levers may influence the use of healthcare services and also improve the supply of services, satisfaction with healthcare services for Armenians partly rests on the increased use of healthcare services - the evidence that of a relatively stronger association between satisfaction with healthcare services and self-perceived health status when compared to the association between satisfaction with healthcare services and the healthcare service use makes the task of improving level of satisfaction with healthcare services by health policy intervention alone more complex. The available datasets do not allow examination of the association of self-perceived health status with discrete dimensions of the health system in a multidimensional construct (for applying targeted measures for health system improvement). Moreover, a high level of dissatisfaction (>60 % for each year) makes it more difficult to find the predictors of satisfaction with precision.

To conclude, our findings suggest that a richer understanding of the relationship between self-perceived health status and satisfaction with the health system requires that distinctions be drawn between the conceptually distinct but empirically related domains of self-perceived health status and unmet expectations in life [66].

## Abbreviations

BBP: Basic Benefit Package
ILCS: Integrated Living Conditions Survey
PCA: Principal Component Analysis
PHCR: Primary Health Care Reform
SAH: Self Assessed (perceived) Health
SES: Socioeconomic strata
SHA: State Health Agency
RESET: Ramsey Regression Equation Specification Error Test
VIF: Variance Inflation Factor

## Footnotes

1 Outpatient contacts per person per year in Armenia are 2–2.5 times lower than those of the CIS countries and new member states of the European Union (Source: Ministry of Health of the Republic of Armenia and Health for All Database, WHO/Europe, 2013).

2 Truncation implies even distribution of SES spread over a narrow range, which makes differentiation between the SES difficult.
